# Rhythm in the Premature Neonate Brain: Very Early Processing of Auditory Beat and Meter

**DOI:** 10.1523/JNEUROSCI.1100-22.2023

**Published:** 2023-04-12

**Authors:** Mohammadreza Edalati, Fabrice Wallois, Javad Safaie, Ghida Ghostine, Guy Kongolo, Laurel J. Trainor, Sahar Moghimi

**Affiliations:** ^1^Institut National de la Santé et de la Recherche Médicale, Unité Mixte de Recherche 1105, Groupe de Recherches sur l'Analyse Multimodale de la Fonction Cérébrale, Université de Picardie, 80054 Amiens, France; ^2^Electrical Engineering Department, Ferdowsi University of Mashhad, 9177948974 Mashhad, Iran; ^3^Institut National de la Santé et de la Recherche Médicale, Unité Mixte de Recherche 1105, Groupe de Recherches sur l'Analyse Multimodale de la Fonction Cérébrale, Explorations Fonctionnelles du Système Nerveux Pédiatriques, Centre Hospitalier Universitaire d'Amiens, 80054 Amiens, France; ^4^Department of Psychology, Neuroscience, and Behaviour, McMaster University, Hamilton, Ontario L8S 3L8, Canada; ^5^McMaster Institute for Music and the Mind, McMaster University, Hamilton, Ontario L8S 3L8, Canada; ^6^Rotman Research Institute, Baycrest Hospital, Toronto, Ontario M6A 2E1, Canada

**Keywords:** electroencephalography, entrainment, frequency tagging, music, phase coupling, premature human brain

## Abstract

The ability to extract rhythmic structure is important for the development of language, music, and social communication. Although previous studies show infants' brains entrain to the periodicities of auditory rhythms and even different metrical interpretations (e.g., groups of two vs three beats) of ambiguous rhythms, whether the premature brain tracks beat and meter frequencies has not been explored previously. We used high-resolution electroencephalography while premature infants (*n* = 19, 5 male; mean age, 32 ± 2.59 weeks gestational age) heard two auditory rhythms in the incubators. We observed selective enhancement of the neural response at both beat- and meter-related frequencies. Further, neural oscillations at the beat and duple (groups of 2) meter were phase aligned with the envelope of the auditory rhythmic stimuli. Comparing the relative power at beat and meter frequencies across stimuli and frequency revealed evidence for selective enhancement of duple meter. This suggests that even at this early stage of development, neural mechanisms for processing auditory rhythms beyond simple sensory coding are present. Our results add to a few previous neuroimaging studies demonstrating discriminative auditory abilities of premature neural networks. Specifically, our results demonstrate the early capacities of the immature neural circuits and networks to code both simple beat and beat grouping (i.e., hierarchical meter) regularities of auditory sequences. Considering the importance of rhythm processing for acquiring language and music, our findings indicate that even before birth, the premature brain is already learning this important aspect of the auditory world in a sophisticated and abstract way.

**SIGNIFICANCE STATEMENT** Processing auditory rhythm is of great neurodevelopmental importance. In an electroencephalography experiment in premature newborns, we found converging evidence that when presented with auditory rhythms, the premature brain encodes multiple periodicities corresponding to beat and beat grouping (meter) frequencies, and even selectively enhances the neural response to meter compared with beat, as in human adults. We also found that the phase of low-frequency neural oscillations aligns to the envelope of the auditory rhythms and that this phenomenon becomes less precise at lower frequencies. These findings demonstrate the initial capacities of the developing brain to code auditory rhythm and the importance of special care to the auditory environment of this vulnerable population during a highly dynamic period of neural development.

## Introduction

Rhythm perception and synchronization to periodicity are cornerstones of language development, music behaviors, bonding, and social interaction early in development (Thomson and Goswami, 2008; Hove and Risen, 2009; Cirelli et al., 2018; Choi et al., 2020; Savage et al., 2021). Rhythmic regularity is ubiquitous in biological systems as it helps organize internal regulation (e.g., heartbeats), locomotion (e.g., walking), perception (e.g., music, language), and thought.

Musical notes and speech phonemes occur in rapid sequences. Further, unlike the case for many visual objects, sounds such as speech and musical tones have a fleeting duration after which they cannot be resampled. It is therefore necessary to organize these sequences into groupings in real time to extract their meanings. Rhythmic structure in speech and music greatly simplifies this problem, making rhythmic perception critical for music and language development. Indeed, behavioral studies show newborns use rhythm to discriminate language categories (Nazzi et al., 1998; Ramus et al., 2000). Although rhythms often contain sound events of different durations, listeners extract a regular or quasi-isochronous pulse or beat, corresponding to how one would tap to the rhythm. Furthermore, listeners perceptually group beats (typically groups of two or three), creating a metrical hierarchy of tempos (Penel and Drake, 1998; Trainor et al., 2009; Nozaradan et al., 2011; Lenc et al., 2020; Møller et al., 2021; Nave-Blodgett et al., 2021; Flaten et al., 2022). Behavioral studies show infants discriminate rhythmic patterns (Hannon and Trainor, 2007); neural evidence suggests late premature newborns are already sensitive to rhythmic temporal patterns (Winkler et al., 2009; Háden et al., 2015; Barajas et al., 2021; Edalati et al., 2022). In adults, listening to rhythmic patterns leads to neural entrainment to beat (Fujioka et al., 2012; Chang et al., 2019) and meter (Nozaradan et al., 2011) periodicities. There is even selective neural enhancement to meter frequencies (Nozaradan et al., 2012b; Lenc et al., 2020). Behavioral studies show infants flexibly perceive metrical cues (Phillips-Silver and Trainor, 2005; Hannon and Trehub, 2005a), and EEG studies show neural entrainment to both the beat and metrical frequencies (Cirelli et al., 2016; Choi et al., 2020; Flaten et al. 2022; Lenc et al. 2022). However, little is known about how early in development infants are able to process rhythms.

As early as 25 weeks gestational age (wGA), structural components of the auditory system allow the fetus to hear the omnipresent rhythms of the maternal heartbeat and respiration (Dunham, 1990; Cheour-Luhtanen et al., 1996) as well as the rhythms of environmental footfalls, speech, and songs (Kisilevsky et al., 2004; Granier-Deferre et al., 2011). The brain undergoes rapid structural and functional development during the third trimester (Kostović et al., 2019, 2021). During this period, the synaptic connections are refined not only by spontaneous activity (Winnubst et al., 2015; Babola et al., 2018; Moghimi et al., 2020; Saadatmehr et al., 2022) but also by sensory-driven neural activity (Minlebaev et al., 2007; Colonnese et al., 2010; Colonnese and Khazipov, 2010; Wess et al., 2017; Moghimi et al., 2020; Saadatmehr et al., 2022). Processing auditory information from the environment requires the development of primary auditory as well as associative cortical networks. Although rapid neural development is ongoing during the third trimester of gestation, fetuses and premature newborns already respond to various auditory stimuli and begin to structure their auditory environment (Mahmoudzadeh et al., 2017; Moser et al., 2021; Edalati et al. 2022). However, there is no neural evidence on the capacities for processing auditory rhythms at this stage of development.

Here, we investigate to what extent rhythm sensitivity is already present in neonates born between 30 and 33 wGA, measuring high-resolution electroencephalography (EEG) in the incubator in the neonatal intensive care unit. In particular, we examine whether premature neonates encode different levels of the metrical hierarchy, as in older infants (Cirelli et al., 2016; Flaten et al., 2022). To compare with 6-month-old infants, we used the repeating six-beat stimulus of Cirelli et al. (2016). This rhythm is ambiguous in containing evidence for both two-beat (three groups of two beats per six-beat pattern) and three-beat (two groups of three beats) groupings. To compare with adults, we presented the 12-beat repeating rhythm stimulus of Nozaradan et al. (2011, 2012a), which has clear four-beat metrical groupings. At this developmental stage, thalamocortical and corticocortical connections are still immature. If premature neonates are only sensitive to the physical properties of sequences, enhanced neural activity should primarily reflect the beat frequency. However, if the premature brain encodes higher-level metrical structures, we should also observe enhanced neural activity at meter-related frequencies.

## Materials and Methods

### Participants

An initial population of 20 healthy premature neonates was decided on in the proposal for obtaining the ethical approvals for this study, based on the previous studies in the laboratory in premature neonates of the same gestational age with auditory stimulation (Mahmoudzadeh et al. 2017; Edalati et al. 2022). One recording was not included in the study because of a system error that resulted in stopping the recording, making the sample size equal to 19 (five males; Table 1), with mean gestational age at birth 32 ± 2.59 wGA (mean recording age, 33.57 ± 2.21 wGA). EEGs were recorded during sleep in incubators at the neonatal intensive care unit of the Amiens University Hospital in France. All neonates had appropriate birth weight, size, and head circumference for their gestational age and normal auditory and clinical neurological assessments. None were considered to be at risk of brain damage. For inclusion, neurological examination results at the time of the recordings had to correspond to the corrected gestational age, with no history of abnormal movements. The gestational age (estimated from the mothers' last period and ultrasound measurements during pregnancy) was consistent with the degree of brain maturation (evaluated on the EEG). The brain imaging results (particularly transfontanellar ultrasound and standard EEG) had to be normal. One or both parents were informed about the study and provided their written informed consent. The local ethics committee (Comité de Protection des Personnes Ouest I) approved the study (ID-RCB: 2019-A01534-53).

**Table 1.** Clinical features of the tested neonates

Neonate number	Sex	GA at birth (week)	GA at test (week)	Birth weight (g)	Apgar (1 min)	Apgar (5 min)	Brain US	EEG Cap	Delivery	Presentation
1	F	33	34	2460	3	7	Normal	Normal	Vaginal	Cephalic
2	M	32	33	1810	10	10	Normal	Normal	Vaginal	Cephalic
3	F	34	35	1550	10	10	Normal	Normal	Cesarean	Cephalic
4	F	32	33	1640	10	10	Normal	Normal	Cesarean	Breech
5	F	32	33	960	6	8	Normal	Normal	Cesarean	Breech
6	M	31	32	1770	10	9	Normal	Normal	Vaginal	Cephalic
7	M	32	34	1540	10	8	Normal	Normal	Cesarean	Cephalic
8	F	33	34	1690	10	10	Normal	Normal	Vaginal	Cephalic
9	F	34	35	2740	5	6	Normal	Normal	Vaginal	Cephalic
10	F	30	31	1475	8	10	Normal	Normal	Vaginal	Breech
11	F	30	31	1370	8	10	Normal	Normal	Vaginal	Breech
12	F	32	32	1760	8	10	Normal	Normal	Cesarean	Cephalic
13	F	32	32	1760	10	10	Normal	Normal	Cesarean	Cephalic
14	F	29	33	1500	2	5	Normal	Normal	Cesarean	Breech
15	F	33	35	2100	6	8	Normal	Normal	Vaginal	Cephalic
16	F	29	35	850	10	10	Normal	Normal	Cesarean	Breech
17	F	29	31	1425	10	10	Normal	Normal	Vaginal	Cephalic
18	M	31	31	1400	5	5	Normal	Normal	Cesarean	Cephalic
19	M	33	36	2280	10	10	Normal	Normal	Vaginal	Cephalic

US, Ultrasonography; F, female; M, male.

### Auditory stimuli and the experimental paradigm

**Figure 1.**
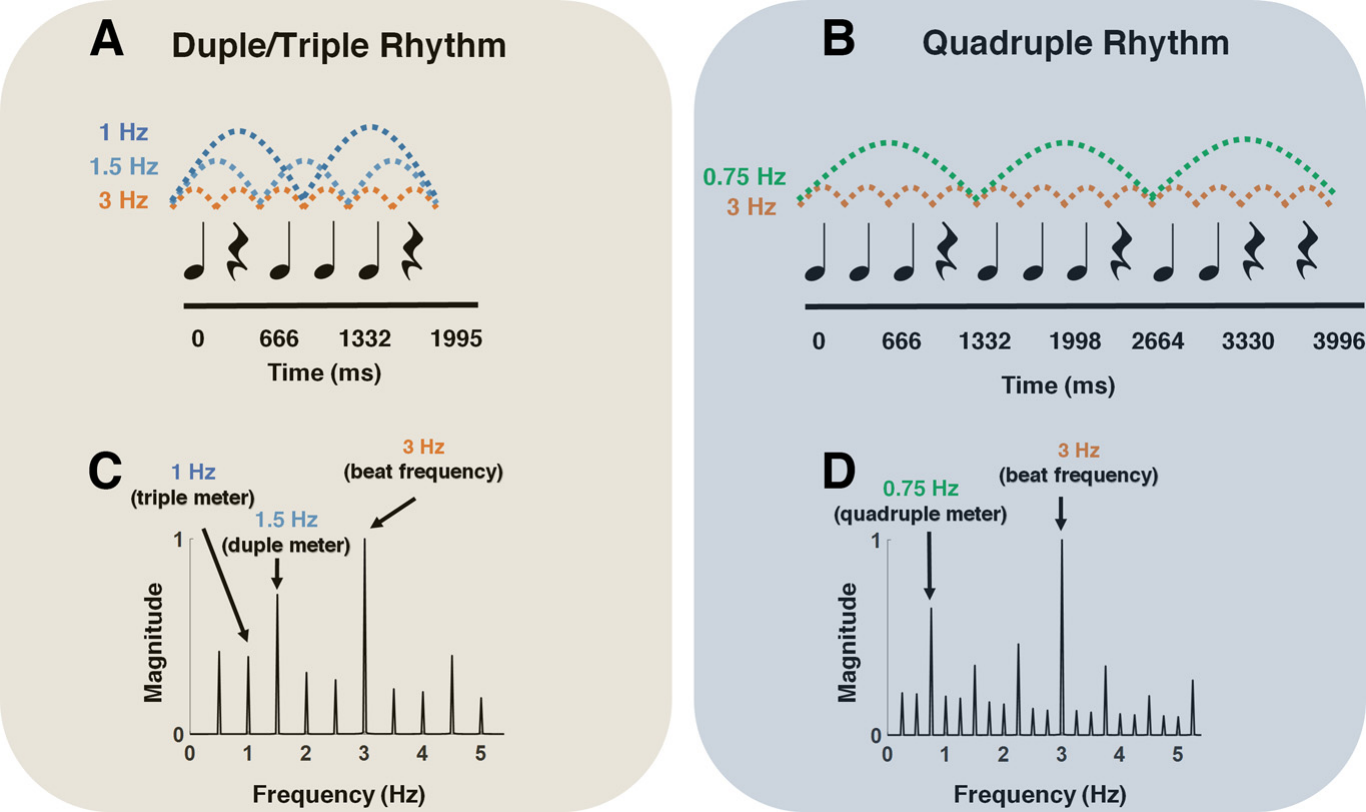
***A***, ***B***, The two rhythmic stimulus patterns used in this study. Both rhythmic patterns consisted of 333-ms-long tones and rests. The dashed lines show the beat and metrical levels. ***C***, ***D***, The frequency spectra of the stimulus sound envelopes.

The stimulus consisted of two rhythmic patterns, named hereafter Duple/Triple Rhythm and Quadruple Rhythm. To create the rhythmic patterns, rock drum sounds composed of snare and bass were used. The stimuli were created using the open-source software Audacity 2.2.2 program and exported as WAV files. Duple/Triple Rhythm consisted of a six-beat rhythmic pattern based on Phillips-Silver and Trainor (2005) and Cirelli et al. (2016) that lasted 2 s (Fig. 1*A*) and was repeated 19 times for 38-s-long trials. Quadruple Rhythm consisted of a 12-beat rhythmic pattern based on Nozaradan et al. (2011, 2012a), that lasted 3.996 s (Fig. 1*B*), and was repeated nine times for 36-s long trials. Each beat in both rhythmic patterns had a 333 ms inter-onset interval (180 beats per minute), which translated into a beat frequency of 3 Hz. The two rhythms were selected based on previous evidence that they induce the perception of a meter, based on grouping by two (duple meter; i.e., 2 × 333 ms = 666 ms = 1.5 Hz) or three beats (triple meter; i.e., 3 × 333 ms = 999 ms = 1 Hz) for Duple/Triple Rhythm (Phillips-Silver and Trainor, 2005; Cirelli et al., 2016), and based on grouping by four beats (quadruple meter; i.e., 4 × 333 ms = 1332 ms = 0.75 Hz) for Quadruple Rhythm (Nozaradan et al., 2011, 2012a). To determine the frequencies at which steady-state evoked potentials were expected to be elicited in the recorded EEG signals, the temporal envelopes of the sounds corresponding to the two rhythms were extracted using the Hilbert transform. The obtained waveforms were then transformed in the frequency domain using a discrete Fourier transform, yielding the frequency spectra of the acoustic energy. As shown in Figure 1, *C* and *D*, the envelopes of both Duple/Triple and Quadruple Rhythms consisted of distinct frequencies ranging from the frequency corresponding to the period of the entire rhythm to the frequencies corresponding to the periods of the grouped (meter) as well as beat levels.

Each experimental session started with 20 min of silence, during which the spontaneous neural activity of the neonate was recorded as a baseline condition for future analysis (data not shown). Next, the trials corresponding to each rhythm were presented to sleeping neonates in separate blocks; four blocks (two for Duple/Triple Rhythm and two for Quadruple Rhythm) were ordered randomly for each participant via loudspeakers placed at the infant's feet. Each block consisted of 25 trials of the rhythm for that block. This made the total number of trials corresponding to each rhythm equal to 50. Block durations of Duple/Triple Rhythm and Quadruple Rhythm were 950 s and 900 s, respectively. The blocks were separated by 30 s of silence. The stimuli were delivered through a speaker at 65 dB SPL, which was located at the feet of the neonates, using Psychtoolbox for MATLAB (Kleiner et al., 2007). The total duration of the experiment was 63 min. The recordings were stopped if the infants woke up, started to cry, or moved.

### EEG acquisition and preprocessing

**Figure 2.**
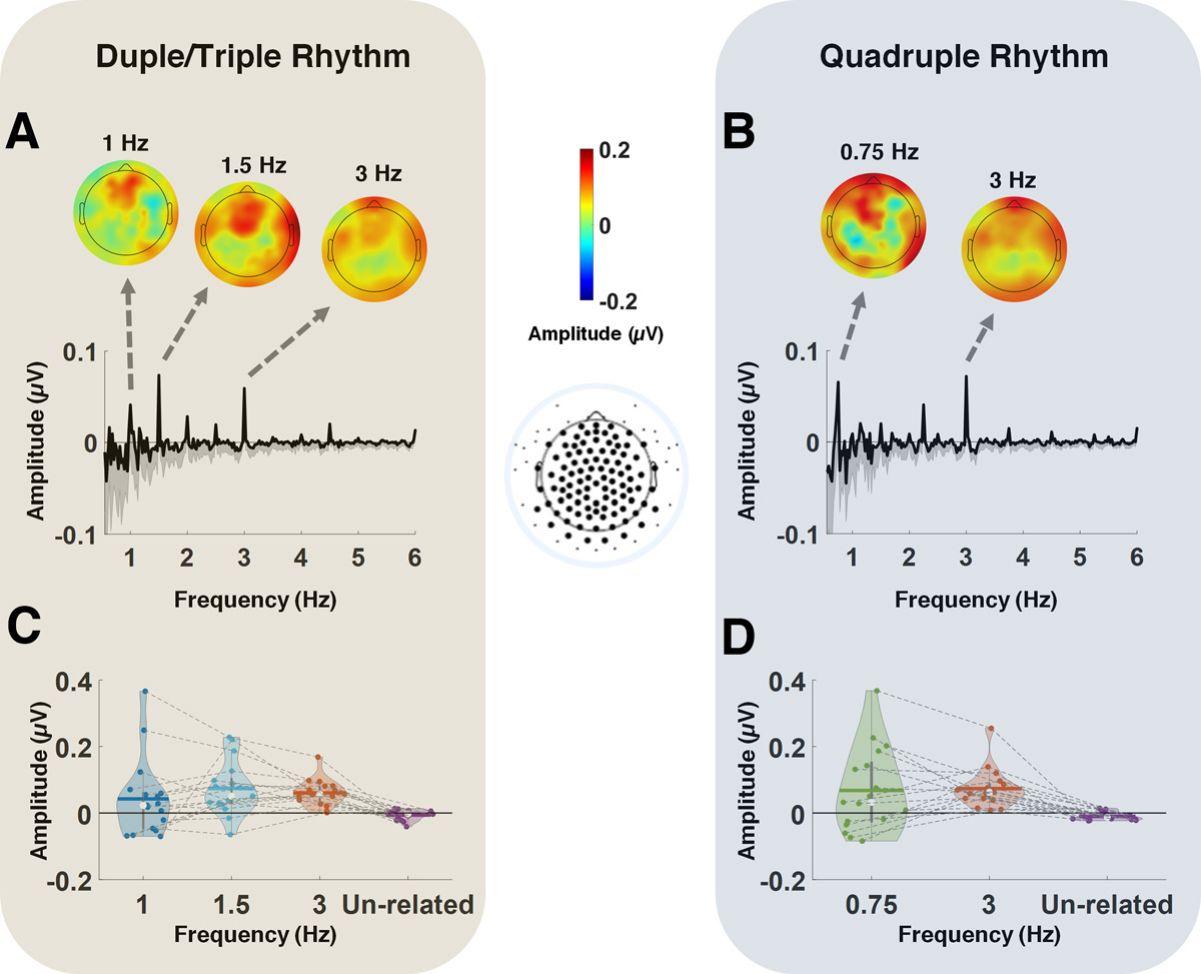
***A***, ***B***, Frequency spectra of the EEG while listening to Duple/Triple Rhythm (***A***) and Quadruple Rhythm (***B***). The values are presented as noise subtracted and averaged across all electrodes (electrodes included in the averaging process, after removing the outer ring, are shown in bold). Top, Topographical maps averaged across participants corresponding to the triple meter frequency (1 Hz), duple meter frequency (1.5 Hz), and beat frequency (3 Hz) for Duple/Triple Rhythm (***A***), and to the quadruple meter frequency (0.75 Hz) and beat frequency (3 Hz) for Quadruple Rhythm (***B***). ***C***, ***D***, Violin plots depict the distribution of individual responses to the beat and meter frequencies as well as the average noise floor, for Duple/Triple Rhythm (***C***) and Quadruple Rhythm (***D***). Lines connect the beat, meter, and unrelated frequency results for each subject. The white dot and the horizontal line indicate the median and mean for each condition, respectively. Paired-samples *t* tests corrected for multiple comparisons showed that the amplitudes of beat- and meter-related frequencies were significantly above the average noise floor. Specifically, *p* = 0.0009 for triple meter frequency (1.5 Hz), *p* = 0.0018 for duple meter frequency (1 Hz), and *p* = 0.0013 for beat frequency (3 Hz), corresponding to Duple/Triple Rhythm, and *p* = 0.0009 for quadruple frequency (0.75 Hz),and *p* = 0.0004 for beat frequency (3 Hz), corresponding to Quadruple Rhythm.

EEG signals were collected using a 124-channel HydroCel Geodesic Sensor Net with an Electrical Geodesic NetAmps 200 amplifier passing a digitized signal to Electrical Geodesics Net Station software (version 5). The EEG was digitized at a 1000 Hz sampling rate, with a Cz vertex electrode as reference. The recorded signals were analyzed with MATLAB software (MathWorks) using FieldTrip (Oostenveld et al., 2011), EEGLAB (Delorme and Makeig, 2004), and custom MATLAB functions and codes. We applied a two-pass 0.5–45 Hz finite impulse response filter (order, 3 cycles of the low-frequency cutoff) and a 50 Hz notch filter by EEGLAB toolbox to remove low- and high-frequency artifacts and also the line noise from the EEG signals. The data were then downsampled to 512 Hz. We removed the electrodes belonging to the outer ring because of their low signal-to-noise ratio (98 electrodes remaining; Fig. 2). The EEG signals were visually inspected, and bad channels and large artifacts and the beginning of acquisition were removed from the data. Next, each channel in each trial was marked and removed from further processing if the average absolute value over the trial exceeded 30 µV. If the number of marked channels in a trial was >50% of all channels, the whole trial was discarded. Two participants were eliminated after this step due to the small number of remaining trials. We corrected the remaining local and transient artifacts, benefiting from our dense electrode layout and the Artifact Blocking algorithm (Fujioka et al., 2011), using a threshold of 100 μV. EEG data were later rereferenced to the average reference.

### Spectral analysis of EEG

We quantified entrainment at the beat and meter frequencies using the frequency-tagging approach (Nozaradan, 2014). EEG signals were first averaged across trials for each participant and rhythmic pattern to improve the signal-to-noise ratio and reduce activities that were non-phase locked to the stimulation train (Nozaradan et al., 2011). The spectra of the steady-state evoked potentials corresponding to the rhythmic patterns were calculated by applying a discrete Fourier transform to the averaged EEG waveforms at each electrode, thereby producing a frequency spectrum with a frequency resolution of 0.028 Hz. The amplitude of each obtained frequency in the spectrum would be expected to correspond to the activity induced by the beat or a meter frequency related to the stimulus, or to correspond to unrelated residual background noise (Nozaradan et al., 2011, 2012a). The contribution of unrelated residual background noise was removed by subtracting the averaged amplitude measured at neighboring frequency bins from each frequency bin (Mouraux et al., 2011; Nozaradan et al., 2011, 2012a; Cirelli et al., 2016). This procedure assumes that the spectrum amplitude at a given frequency bin is similar to the spectrum amplitude of the mean of the surrounding frequency bins in the absence of a steady-state evoked potential (Nozaradan et al., 2011). The neighboring bins ranged from −0.15 to −0.07 Hz and +0.07 to +0.15 Hz (−3 to −5 and +3 to +5 bins around each frequency bin).

### Brain-stimulus synchronization

To evaluate whether the brain successfully synchronized to the rhythmic sequences at beat and meter frequencies, we calculated an index that quantified brain-stimulus synchronization. For this, the time-frequency information for the stimulus rhythmic patterns and separate EEG trials was calculated using Morlet wavelet transformation (Morlet wavelets consisting of seven cycles) in 2 ms steps (0.25 Hz resolution). After removing the first and last 2 s (for Duple/Triple Rhythm) and the first and last 4 s (for Quadruple Rhythm), the obtained complex wavelet coefficients were normalized for the magnitudes. The phase times series of averaged complex wavelet coefficients corresponding to EEG ($\theta_{EEG}\left(t,f\right)$) and rhythmic patterns ($\theta_{Rhyt}\left(t,f\right)$) were extracted at beat and meter-related frequencies (1, 1.5, and 3 Hz for Duple/Triple Rhythm, and 0.75 and 3 Hz for Quadruple Rhythm) and averaged over time to calculate a synchronization index (Ghuman et al., 2011; Assaneo and Poeppel, 2018; Orpella et al., 2022) as follows:

SI(f)=1N∑t=1Ne j[θEEG(t,f)−θRhyt(t,f)]

SI is a complex value in which the absolute value and angle represent the strength of locking and the phase difference between the neural activity and stimulus rhythms, respectively, at different frequencies. The SI was calculated at each electrode location for each participant.

### Statistical analysis

Statistical analyses were performed with MATLAB (MathWorks), using the FieldTrip (Oostenveld et al. 2011) and CircStat (Berens, 2009) toolboxes as well as custom MATLAB functions.

#### Testing neural responses to beat and meter frequencies

For the spectral analysis of EEG, the spectra were averaged across all scalp electrodes (Fig. 2) for each participant to avoid any electrode selection bias (Nozaradan et al., 2011, 2015; Cirelli et al., 2016; Nozaradan et al., 2016). Paired-samples *t* tests, corrected for multiple comparisons using Bonferroni correction, were used to determine whether the obtained amplitudes measured at the beat and meter frequencies were significantly different from the average noise floor (mean amplitude of frequencies unrelated to the beat and meter). The effect size was defined using Cohen's *d*.

#### Testing neural enhancement of meter frequencies

To evaluate the possible neural enhancement of specific frequencies corresponding to meter in the EEG responses, we normalized the amplitude of the meter frequencies (1 and 1.5 Hz for Duple/Triple Rhythm and 0.75 Hz for Quadruple Rhythm) by that of the beat frequency (3 Hz) for the spectra corresponding to both the rhythmic patterns and EEG responses for each participant. Once normalized, we compared the amplitude of the neural response with that of the rhythmic stimuli at meter-related frequencies using a paired-samples *t* test. The corresponding effect size was defined using Cohen's *d*.

#### Testing brain-stimulus synchronization

To evaluate the consistency of phase synchronization in brain-stimulus synchronization for beat, duple, triple and quadruple meters, we performed the basic Rayleigh test (Berens, 2009) to evaluate the nonuniformity of the circular histogram of the SI angle over participants, which provides a measure of consistent phase locking to the rhythmic sequence.

### Data availability

The stimuli and data that support the findings of this study are available on reasonable request from the corresponding authors (S.M. and L.J.T). The data are not publicly available because of participants not providing consent to share their data outside our research consortium on the consent form. MATLAB code and data matrices are available on GitHub (https://github.com/mredalati/RhythmPretermNeonates.git).

## Results

### Spectral analysis of EEG

#### Do premature infants show neural responses to beat and meter frequencies?

The spectra of the steady-state evoked potential responses corresponding to the two rhythmic patterns, averaged across all electrodes, are depicted in Figure 2, *A* and *B*. As illustrated, both Duple/Triple Rhythm and Quadruple Rhythm elicited frequency-tagged EEG responses. The expected beat frequency was 3 Hz (i.e., 333 ms long tones and rests) for both rhythmic patterns. For Duple/Triple Rhythm, the expected metrical frequencies were 1.5 and 1 Hz, corresponding to where beats are grouped by two (duple) and three (triple) beats, respectively (Fig. 2*A*). For Quadruple Rhythm, 0.75 Hz represents the expected metrical frequency where beats are grouped by four (quadruple; Fig. 2*B*). Topographical scalp distributions of neural responses corresponding to each of the aforementioned frequencies are also presented, showing similar topographical distributions for the two rhythmic patterns (Duple/Triple and Quadruple) for both beat and meter frequencies.

To evaluate the significance of the frequency-tagging response, first, the amplitudes in the EEG spectra (averaged over electrodes), corresponding to the two rhythmic patterns, were calculated at frequencies where peaks were observed in the sound stimulus (1, 1.5, 2, 2.5, and 3 Hz for Duple/Triple Rhythm; 0.75, 1, 1.25, 1.5, 1.75, 2, 2.25, 2.5, 2.75, and 3 Hz for Quadruple Rhythm; 0.25 and 0.5 Hz were excluded because we used a 0.5 Hz high-pass filter). The calculation was repeated at frequencies with no peaks in the two stimuli (0.75, 1.25, 1.75, 2.25, and 2.75 Hz for Duple/Triple Rhythm; 0.625, 0.875, 1.125, 1.375, 1.625, 1.875, 2.125, 2.375, 2.625, and 2.875 Hz for Quadruple Rhythm). Average noise floor amplitude for each rhythm pattern was calculated as the average across frequencies in the latter groups. Figure 2, *C* and *D*, depict the violin plots corresponding to the individual frequency-tagging responses at the beat and meter frequencies for each of the two rhythmic patterns, as well as the frequencies unrelated to beat and meter (average noise floor). Visual inspection of these figures reveals that the number of participants with a frequency-tagging response equal to/below the noise floor was smaller for the duple meter frequency compared with either the triple meter in Duple/Triple Rhythm or the quadruple meter in Quadruple Rhythm. Nevertheless, spectra amplitudes at each of the beat and meter frequencies analyzed were significantly above the average noise floor at the group level (*t*_(16)_ = 3.46, p = 0.0009, Cohen's *d* = 0.79 for triple meter frequency; *t*_(16)_ = 3.23, *p* = 0.0018, Cohen's *d* = 1.22 for duple meter frequency; and *t*_(16)_ = 3.34, *p* = 0.0013, Cohen's *d* = 1.94 for beat frequency in Duple/Triple Rhythm; *t*_(16)_ = 3.38 *p* = 0.0009, Cohen's *d* = 0.68 for quadruple meter frequency; *t*_(16)_ = 3.65, *p* = 0.0004, Cohen's *d* = 2.16 for beat frequency in Quadruple Rhythm), as verified by paired-samples *t* tests corrected for multiple comparisons (Bonferroni correction). No other amplitudes at frequencies present in the stimulus were significantly larger than the noise floor value. Thus, neural responses to beat, duple, triple, and quadruple meter frequencies are present already in the premature brain.

#### Are neural responses to meter frequencies enhanced compared with beat frequencies?

**Figure 3.**
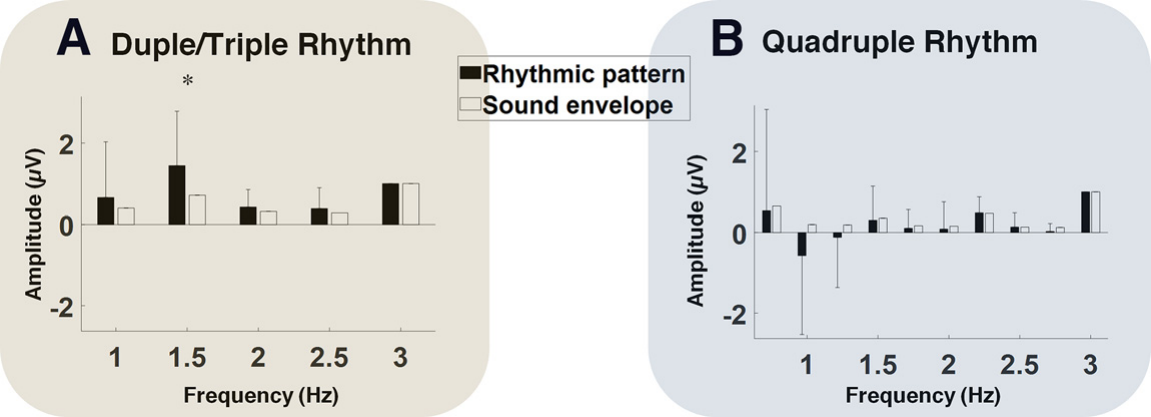
***A***, ***B***, Comparison of frequency spectra of stimulus and EEG rhythmic patterns for Duple/Triple Rhythm (***A***) and Quadruple Rhythm (***B***). The amplitude values are normalized by the amplitude at beat frequency (3 Hz). A paired-samples *t* test showed that the amplitude of the spectrum corresponding to Duple/Triple Rhythm at duple meter frequency (1.5 Hz) was significantly larger than the amplitude of the spectrum of the stimulus sound envelope at this frequency (*t*_(16)_ = 2.17, *p* = 0.0461, Cohen's *d* = 0.75). There were no significant differences at any other frequencies. For the Quadruple Rhythm, there was no significant difference between the stimulus and EEG amplitudes at 1.5 Hz or any other frequencies.

Visual comparison of the spectra corresponding to the stimuli (Fig. 1*C*) and the EEG responses (Fig. 2*A*,*B*) demonstrated an enhancement of the neural response at meter-related frequencies, with respect to the neural response at beat frequency. More precisely, the amplitudes at the meter-related frequencies were smaller than those of the beats in the stimuli spectra, whereas they were larger than those of the beat in the EEG response. To quantitively evaluate the possible enhancement of neural response to meter-related frequencies we compared the normalized spectra (Fig. 3) corresponding to the EEG responses and rhythmic stimuli. The amplitude of the spectrum corresponding to the neural response to Duple/Triple Rhythm at the duple meter frequency (1.5 Hz) was significantly larger than the amplitude of the duple meter frequency in the spectra corresponding to the envelope of Duple/Triple Rhythm (*t*_(16)_ = 2.17, *p* = 0.0461, Cohen's *d* = 0.75). There was no significant difference between the spectra of neural responses and those of the rhythmic patterns at triple and quadruple meter frequencies for either of the rhythmic patterns (Fig. 3).

### Brain-stimulus synchronization

**Figure 4.**
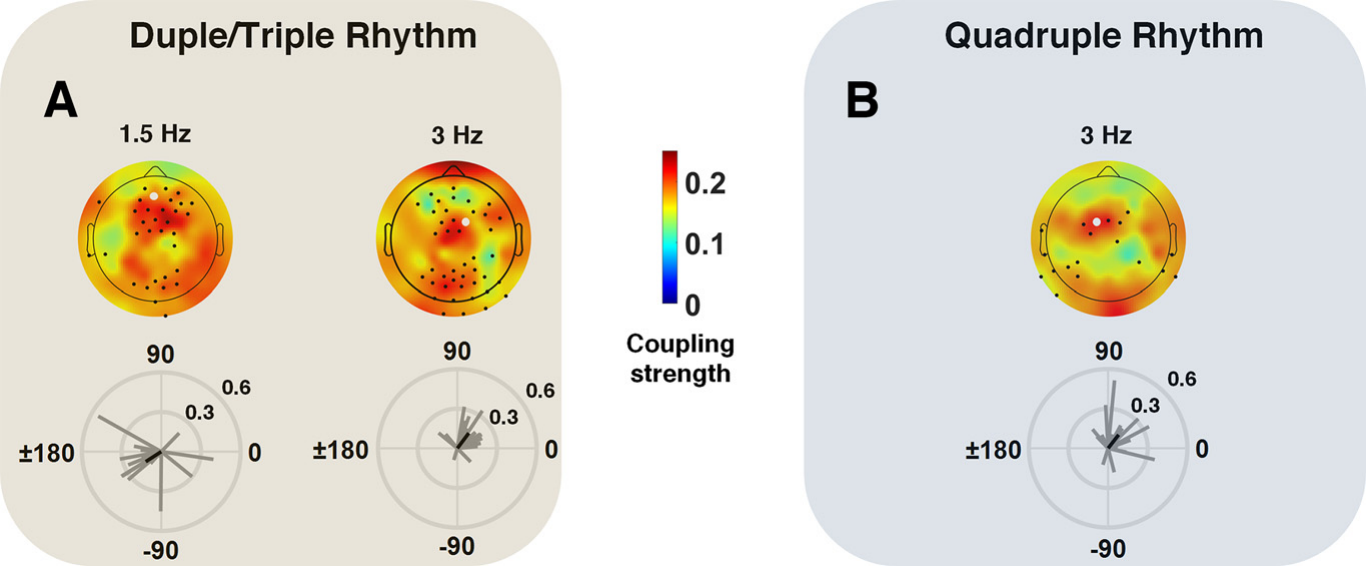
***A***, ***B***, The topographical maps of averaged coupling strength between neural activity and rhythmic patterns are shown for Duple/Triple Rhythm (***A***) and Quadruple Rhythm (***B***). The electrodes for which circular nonuniformity of phase values was confirmed using the Rayleigh test (*p* < 0.05) are indicated by black dots. The individual coupling phase and strength are shown for sample electrodes (white dots) below the topographical maps. The black vector illustrates the circular group average.

To address direct synchronization from rhythmic stimuli to brain we evaluated the angle of the defined SI, at each electrode location, for all participants. The phase lag between neural activity and rhythmic patterns was consistent among participants over a number of electrodes for beat frequencies corresponding to Duple/Triple and Quadruple Rhythm. This consistency existed also over the frequency corresponding to duple meter (1.5 Hz), but not over the frequencies corresponding to triple (1 Hz) and quadruple (0.75 Hz) meters. Figure 4, *A* and *B*, illustrate the topographical distribution of mean coupling strength and the distribution of phase lag over selected electrodes for all participants, corresponding to Duple/Triple and Quadruple Rhythm. Electrodes with a phase lag distribution significantly different from the uniform distribution (hence, consistent phase locking between neural activity and auditory stimulation, as verified by the Rayleigh test, *p* < 0.05) are marked. As shown by the mean lag vector as well as the phase lag pattern among participants over sample electrodes, the synchronization lag for the beat frequency was replicable from Duple/Triple Rhythm (mean phase value over a selected electrode = 48.98°) to Quadruple Rhythm (mean phase value = 62.33°). The phase lag was larger (mean phase value = −129.74°) for the duple meter compared with the beat, demonstrating synchronization of the neural activity to the duple metrical level of the rhythmic pattern, with a larger time delay than for the beat.

## Discussion

The present study indicates that like in adults the brains of premature infants tested ∼32 wGA entrain to periodicities in rhythmic auditory streams at different metrical hierarchical levels. Specifically, the amplitude of neural activity measured by EEG was significantly greater at the beat frequency and the three tested meter-related frequencies (duple, triple, quadruple) compared with unrelated frequencies. Additionally, oscillatory activity at beat- and duple meter-related frequencies was aligned with the auditory stream, as indicated by significant phase locking between the EEG and rhythmic sequences.

Adults show selective enhancement of neural activity at meter-related compared with beat-related frequencies (Nozaradan et al., 2012b, 2018a, b), even after degradation of acoustic cues guiding meter perception (Lenc et al., 2020), suggesting the adult brain enhances higher-level metrical representations rather than faithfully representing the incoming periodic information. Interestingly, we found evidence consistent with selective enhancement in premature infants for duple meter over the beat frequency (Fig. 3), which could enhance infants' ability to learn meaningful hierarchical units in the rapid sequences of sounds that make up music and language. Although enhancements at triple (1 Hz) and quadruple (0.75 Hz) meter levels were not significant in our premature infant data. This may be because of difficulty processing slow tempos as studies show that the optimal frequency for rhythmic entrainment is ∼1.7–2.0 Hz (500–600 ms onset to onset), and rhythms at tempos slower than 1 are perceived less well by both post-term infants and adults (Fraisse, 1982; Parncutt, 1994; Baruch and Drake, 1997; Van Noorden and Moelants, 1999; Toiviainen and Snyder, 2003), rather than a lack of enhancement of triple and quadruple metrical levels at this age. In adult studies showing enhanced quadruple meter, the quadruple meter was presented at 1.25 Hz, which is much closer to the optimal 1.7 Hz, and considerably faster than for our stimuli (0.75 Hz). Thus, we are unable to ascertain whether the triple and quadruple meter levels were too slow for premature infants to process well or whether early metrical enhancement is largest for duple meter, so this remains for future research.

The duple meter enhancement is intriguing because it requires internal neural representation to interact with incoming sensory information in the premature brain, similarly as shown previously in older infants (Choi et al., 2020; Flaten et al., 2022). It appears that the responses at meter frequencies do not simply reflect a general modulation transfer function between frequency and brain responses as, for example, the normalized power 1.5 Hz is enhanced compared with the stimulus only for Duple/Triple Rhythm and not for Quadruple Rhythm. However, to provide a solid conclusion on the underlying neural mechanism, further control studies are required, where, for instance, the frequency corresponding to duple meter is either degraded in the stimulus or presented in a sequence where it does not correspond to a grouping of two (duple meter). This would allow further determination on whether neural responses observed during rhythmic stimulation truly reflect an endogenous representation of rhythmic/metric information or rather reflect coding of the exogenous structure of the auditory input. That infants at 32 wGA process beat- and meter-related frequencies and show enhancement for at least duple meter-related frequencies (suggesting endogenous processing) is particularly intriguing as it demonstrates sophisticated computational capacities despite the immaturity of cortical and subcortical structures.

At this stage of neurodevelopment, the microstructures of the cortical columns undergo rapid evolution in an inside-out manner (Rakic, 1988), with neurons initially in place in deep layers. Before 26 wGA, thalamocortical afferents accumulate in the superficial subplate. Between 26 and 28 wGA, thalamocortical afferents invade the cortical plate of corresponding target areas, within which the first synapses appear. Between 28 and 30 wGA, thalamocortical axons establish synapses with cortical plate layer IV neurons and become (at least partly) functionally sensory-driven (Kostović and Judaš, 2010; Molnár et al., 2019). This period is also marked by transient neural circuits as well as guidance, waiting, and target selection of thalamocortical and cortico-cortical pathways by the subplate compartments, whose contribution to the evoked activity in infants is yet to be explored (Kostović, 2020).

Our results show that these transient neural circuits are sophisticated in following multiple periodicities in an auditory input and selectively enhancing certain metrical levels. Our findings are in line with recent evidence proposing the existence of more elaborate functional development at this period of gestation than previously assumed. For example, neural responses to violations of global rules structuring auditory sequences cannot be explained by simple bottom-up processing (Moser et al., 2021). Such early functional activities might be guided by the subplate transient circuitry and its tangential nexus across the hemispheres, serving as an alternative transient associative interareal nexus (Kostović, 2020), encompassing large-scale interactions between associative areas within and between hemispheres involving multiple contacts between subplate neurons, interstitial neurons, neurons in migration, and multiple axonal branches of the growing pathways. That early computational capacities are performed by this transient network could partly explain differences in neural responses to acoustic features in prematurity (Mahmoudzadeh et al., 2017; Edalati et al., 2022) from those in older infants. For example, early responses to violations of predictions or expectations set up by auditory patterns manifest in EEG as slow scalp-frontal positivity early in development but as a more rapid negative component (the mismatch negativity) in older infants (Dehaene-Lambertz, 2000; He et al., 2007, 2009; He and Trainor, 2009; Trainor, 2012; Dehaene-Lambertz and Spelke, 2015). This shift might relate to the remodeling of the initial circuitry and the development of more mature neural architectures. This process may also demonstrate that early scalp-measured responses reflect deep sources, whereas later scalp-measured responses reflect more superficial sources, involving middle- and upper-layer circuitry within the cortical plate.

With respect to the alignment between neural responses and the rhythmic stimuli, we found the highest synchronization values at beat and duple meter frequencies. Neural oscillations to metrical cues at relatively slower frequencies (triple and quadruple meters) were less precise. This superior synchronization with the stimulus at duple compared with triple and quadruple meters is consistent with our finding, discussed above, of a larger neural response at the duple meter frequency than expected given the composition of the stimulus, whereas this was not the case for triple and quadruple meter frequencies. Both findings might reflect early superior processing of duple over triple and quadruple meters, but it most likely relates simply to the slower tempi of the triple (1 Hz) and quadruple (0.75 Hz) meters compared with the duple (1.5 Hz) and beat (3 Hz) frequency for the following reasons. First, even in adults, synchronization of the phase of neural activity to presented rhythms falls off at slower tempos (<1 Hz; Doelling and Poeppel, 2015; Doelling et al., 2019). Second, although Western adults preferentially process duple over more complex meters (Povel, 1981; Essens and Povel, 1985), this is largely influenced by experience. Western music has predominantly duple meters, but many musical systems around the world use more complex meters (Snyder et al., 2006; Polak, 2010; Hannon et al., 2012). Although there is a general bias for small-integer ratio metrical groupings, there is considerable variability across cultures (Jacoby and McDermott, 2017), suggesting the human brain has considerable flexibility for meter perception. Third, behavioral studies in Western full-term infants indicate that infants are initially adept at processing both simple and more complex meters, but they lose the ability to process complex meters by 12 months of age if they are not exposed to them (Hannon and Trehub,2005a,b; Hannon and Trainor, 2007), which is similar to how infants' speech-sound categories become specialized for the language in their environment (Werker and Tees, 1999; Kuhl et al., 2006). Furthermore, 6-month-old infants can perceive six-beat repeating rhythm patterns as having either duple or triple meter depending on priming by either movement (being bounced) at the meter frequency (Phillips-Silver and Trainor, 2005) or with loudness accents on every second versus third beat of the rhythm pattern (Flaten et al., 2022).

An important question concerns whether the abilities of premature infants at 32 wGA to process both beat and meter, and selectively enhance at least duple meter frequencies, relies primarily on the genetically driven rapid evolution of auditory neural circuits during the last trimester, or whether experience with auditory patterns is necessary. There is much evidence for experience-dependent maturation during the first year after full-term birth. There is also evidence that newborns remember music and rhymes they were exposed to *in utero* (Partanen et al., 2013). Musical experience has also been associated with enhanced neural responses in infants at meter frequencies (Cirelli et al., 2016; Flaten et al., 2022) and better detection of meter violations (Zhao and Kuhl, 2016). Together, this suggests auditory experience affects neural development from the earliest stages of auditory functioning. Furthermore, in the visual domain, a lack of bottom-up sensory input leads to less developed top-down connections, again suggesting the importance of experience for optimal circuit development (Ibrahim et al., 2021).

If experience with auditory rhythms is important during the last prenatal trimester, then infants born prematurely should benefit from exposure to auditory rhythmic sequences in the Neonatal Intensive Care Unit. Indeed, rhythm and timing deficits are associated with a variety of neurodevelopmental disorders (Trainor et al., 2018; Chang et al., 2021; Lense et al., 2021), suggesting that early evaluation of atypical rhythm-processing capacities could help to identify infants and children at risk for speech and language neurodevelopmental disorders (Ladányi et al., 2020). Our results show considerable individual differences (Fig. 2), with some infants showing particularly poor entrainment despite similar developmental and clinical evaluations as other infants. It is therefore important for future work to assess individual-level associations between rhythmic neural entrainment and additional preterm neurobiomarkers, such as spontaneous neural activity in the perisylvian areas (Saadatmehr et al., 2022), as well as to follow premature infants longitudinally to assess long-term outcomes and the predictive value of very early evaluation of neural rhythm processing in this at-risk population.

One limitation of our study was its relatively small sample size because of difficulties recruiting this population and the inclusion requirements. Future studies should replicate these results and follow a larger cohort developmentally. In additional, future comparison with full-term-born newborns would allow us to address the impact of premature birth on auditory rhythm processing as well as the evolution of the neural response during the third trimester of gestation.
